# Long-term memory requires sequential protein synthesis in three subsets of mushroom body output neurons in *Drosophila*

**DOI:** 10.1038/s41598-017-07600-2

**Published:** 2017-08-02

**Authors:** Jie-Kai Wu, Chu-Yi Tai, Kuan-Lin Feng, Shiu-Ling Chen, Chun-Chao Chen, Ann-Shyn Chiang

**Affiliations:** 1 Institute of Systems Neuroscience, National Tsing Hua University, Hsinchu, 30013 Taiwan; 2 Brain Research Center, National Tsing Hua University, Hsinchu, 30013 Taiwan; 3 Institue of Physics, Academia Sinica, Nankang, Taipei, 11529 Taiwan; 4 Kavli Institute for Brain and Mind, University of California, San Diego, La Jolla, CA 92093-0526 USA

## Abstract

Creating long-term memory (LTM) requires new protein synthesis to stabilize learning-induced synaptic changes in the brain. In the fruit fly, *Drosophila melanogaster*, aversive olfactory learning forms several phases of labile memory to associate an odor with coincident punishment in the mushroom body (MB). It remains unclear how the brain consolidates early labile memory into LTM. Here, we survey 183 *Gal4* lines containing almost all 21 distinct types of MB output neurons (MBONs) and show that sequential synthesis of learning-induced proteins occurs at three types of MBONs. Downregulation of oo18 RNA-binding proteins (ORBs) in any of these MBONs impaired LTM. And, neurotransmission outputs from these MBONs are all required during LTM retrieval. Together, these results suggest an LTM consolidation model in which transient neural activities of early labile memory in the MB are consolidated into stable LTM at a few postsynaptic MBONs through sequential ORB-regulated local protein synthesis.

## Introduction

Memory consolidation stabilizes short-lasting and labile memory into long-lasting and stable memory, which allows animals to behave appropriately when facing the experienced event again in the future^1^. Long-term memory (LTM) formation requires two different levels of consolidation: (*i*) cellular consolidation triggers new protein synthesis and signal transduction cascades to stabilize stimulus-induced specific synapse changes within neurons, and (*ii*) system consolidation reorganizes and stores dynamic information of neural circuit activity throughout multiple brain regions^2–6^. However, how cellular consolidation transfers and integrates dynamic memory information into system consolidation remains unclear. Here, taking advantage of the well-studied aversive olfactory memory in *Drosophila*, we start to delineate memory-encoding circuits (or memory engrams)^4, 7, 8^ by identifying individual brain neurons synthesizing new proteins necessary for LTM at specific time points after learning.

Forming aversive LTM in *Drosophila* requires repetitive experiences with spaced resting intervals and new protein synthesis, similar to that seen in mammals^9–13^. In flies, ten sessions of (10X) spaced training induce robust week-lasting LTM that is sensitive to a protein synthesis inhibitor. In contrast, 10X massed training without interval resting produces a day-lasting intermediate form of anesthesia-resistant memory (ARM) that is insensitive to a protein synthesis inhibitor^10^. New learning-induced proteins rely mainly on two cellular mechanisms to stabilize synaptic plasticity^2, 6, 14^: (*i*) nucleus transcription regulated by the cAMP response element-binding protein (CREB)-dependent transcriptional cascades^15, 16^, and (*ii*) synapse-specific local translation regulated by the oo18 RNA-binding protein (ORB) in *Drosophila*, analogous to the cytoplasmic polyadenylation element-binding protein (CPEB) in mammals^17–20^. In flies, aversive olfactory LTM formation requires CREB- and ORB-dependent protein synthesis in the single dorsal-anterior-lateral (DAL) neurons^21^ and the two mushroom body output neurons (MBON-α3, previously called MB-V3)^22^, respectively. Moreover, ORB2 proteins, which are intrinsically present in KCs of the MB, are essential to enable courtship behavior LTM formation^20, 23, 24^.

Using a particular odor as the conditioned stimulus (CS) and coincident electrical shock as the unconditioned stimulus (US), classical conditioning trains flies to avoid a certain odor as an aversive olfactory memory^25^. The CS inputs received by olfactory sensory neurons are delivered via antennal lobe projection neurons to the MB, which is composed of approximately 2200 Kenyon cells (KCs), extending their axonal fibers anteriorly to form three major presynaptic lobes: α/β, α′/β′, and γ^26^. An odor activates only a sparse number of KCs forming an internal neural coding^27^.

A diverse population of dopaminergic MB input neurons (MBINs) sending axonal terminals subdivides each MB along all lobes into 15 distinct compartments^28^. Artificial activation of specific MBINs mimics the US inputs for different phases of memory, suggesting that CS/US association occurs at specific MB compartments^29–32^. On the other hand, there are at least 21 distinct types of MBONs, presumably relaying processed information in the MB to the downstream higher brain centers^28^. Blocking new protein synthesis in the MBON-α3 after spaced training impaired LTM^22^. Importantly, neurotransmission outputs from MBON-α3 and MBON-V2 are necessary during LTM retrieval^22, 33, 34^.

Recent studies showed that activation of the upstream MBINs with coincident CS delivery modifies the response of downstream MBONs to the CS, and the modified activity of MBONs is independent of neurotransmission release^31, 35, 36^. This functional plasticity induced by learning suggests possible LTM-encoding sites at specific KC-MBON postsynapses. Here, we investigate whether LTM formation requires new learning-induced protein synthesis in other types of MBONs. And, are ORB proteins also required in these MBONs? We found that LTM formation requires sequential protein synthesis in three distinct types of MBONs, each of which connects with one of the three major classes of KCs. Notably, these MBONs all use ORB proteins in LTM formation.

## Results

### MBON-γ3,γ3β′1 and MBON-β′2mp synthesize new proteins necessary for LTM

**Figure 1:**
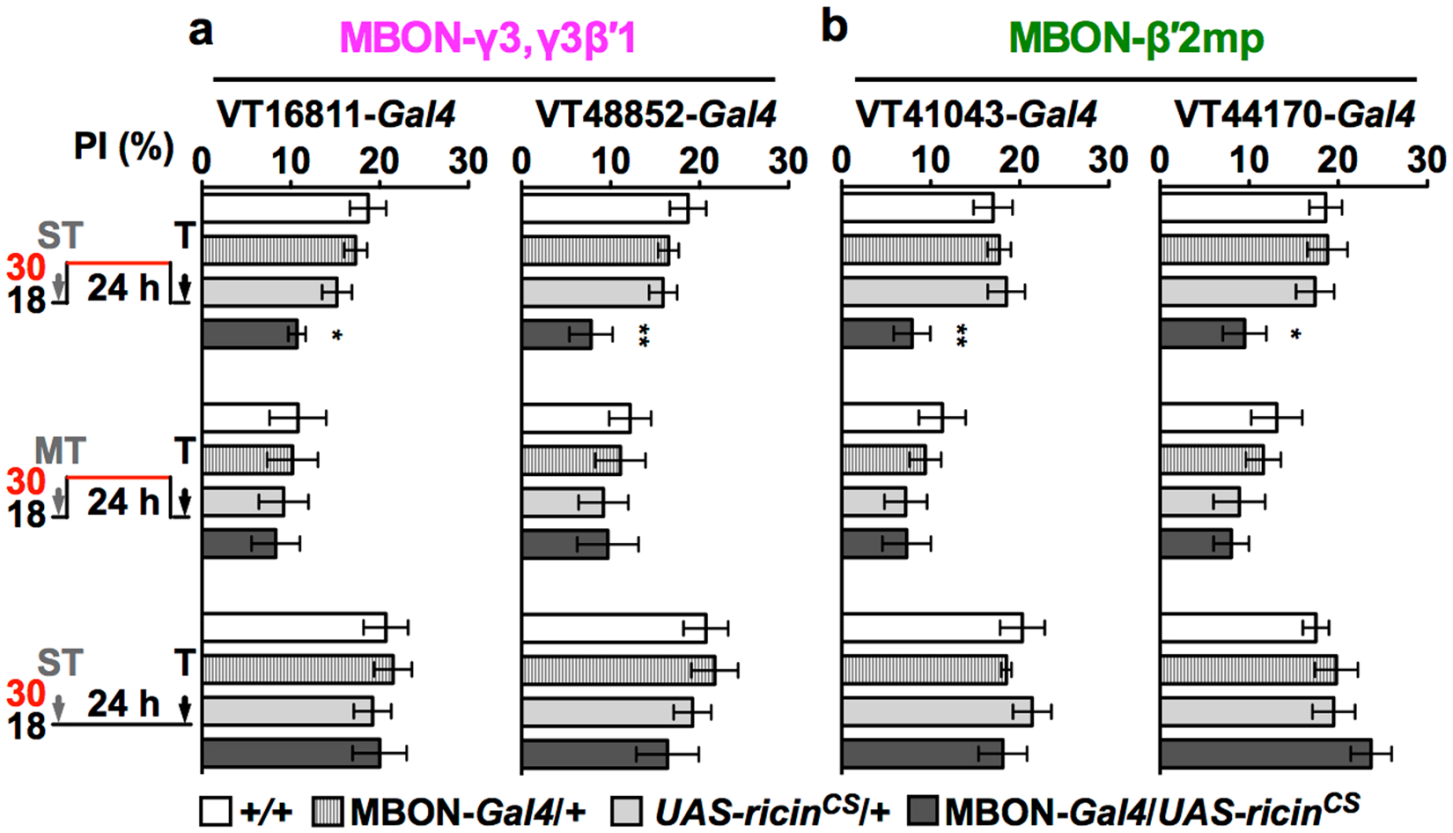
Protein synthesis requirement of LTM in MBON-γ3,γ3β′1 (magenta) or MBON-β′2mp (green). Blocking protein synthesis in MBON-γ3,γ3β′1 (**a**) or MBON-β′2mp (**b**) specifically impaired LTM, but not ARM. Flies were shifted to 30 °C to activate RICIN^CS^ immediately after training (ST: spaced training; MT: massed training) and then shifted back to 18 °C to inactivate RICIN^CS^ 40 min before testing (T). All experiments were tested 24 h after training. Driver alone (VT-*Gal4*/+) or effector alone (*UAS*-*ricin* ^*CS*^/+) did not affect LTM under the same treatment compared with the wild-type control (+/+). Flies of all genotypes under a permissive temperature exhibited normal LTM. Each value = mean ± SEM (n ≥ 8). *p < 0.05, **p < 0.01.

To comprehensively understand LTM formation at the cellular level, we surveyed the requirement of learning-induced protein synthesis by using a reversible cold-sensitive 28S ribosomal RNA cleavage toxin^37–40^, RICIN^CS^, expressed in discrete MBONs in 183 *Gal4* lines after spaced training (Supplementary Table 1). Flies were trained and then tested at a permissive temperature (18 °C) in which RICIN^CS^ was inactive, to avoid interference with memory acquisition and retrieval, but shifted to a restrictive temperature (30 °C to activate RICIN^CS^) to block protein synthesis during memory consolidation. We found that blocking protein synthesis in MBON-γ3,γ3β′1 or MBON-β′2mp impaired 24-h LTM after spaced training, that induced protein-synthesis-dependent LTM (Fig. 1a,b, heat-shock ST), but not after massed training, that induced only protein-synthesis-independent ARM (Fig. 1a,b, heat-shock MT). We confirmed the requirement of new proteins for LTM with additional *Gal4* drivers also expressed at the target neurons (Supplementary Fig. 1a,b). As a positive control, RICIN^CS^ inhibition on MBON-α3 also impaired 24-h memory (Supplementary Fig. 1c,d), as reported previously^22^. As negative controls, spaced-training-induced LTM was normal after RICIN^CS^ inhibition in MBON-V2 cluster, MBON-γ5β′2a, and MBON-β2β′2a (Supplementary Fig. 1a,b). Permissive temperature control showed that inactive RICIN^CS^ did not affect the learning ability or the normal physiology of MBON-γ3,γ3β′1 and MBON-β′2mp (Fig. 1, non-heat-shock ST). To rule out possible effects of RICIN^CS^ via other *Gal4*-positive neurons, we confirmed these results using split-*Gal4* drivers^28, 34^ with intersected expression only in MBON-γ3,γ3β′1, MBON-β′2mp, or MBON-α3 (Supplementary Fig. 1c,d). We also found that 24-h blocking of protein synthesis in these three types of MBONs prior to 1X learning did not affect immediate memory, suggesting a normal CS/US association (Supplementary Fig. 2g–i). Thus, our data indicated that normal LTM formation requires spaced-training-induced protein synthesis in three distinct types of MBONs: MBON-α3, MBON-γ3,γ3β′1, and MBON-β′2mp.

### Sequential protein synthesis in MBON-α3, MBON-γ3,γ3β′1, and MBON-β′2mp consolidates LTM

**Figure 2:**
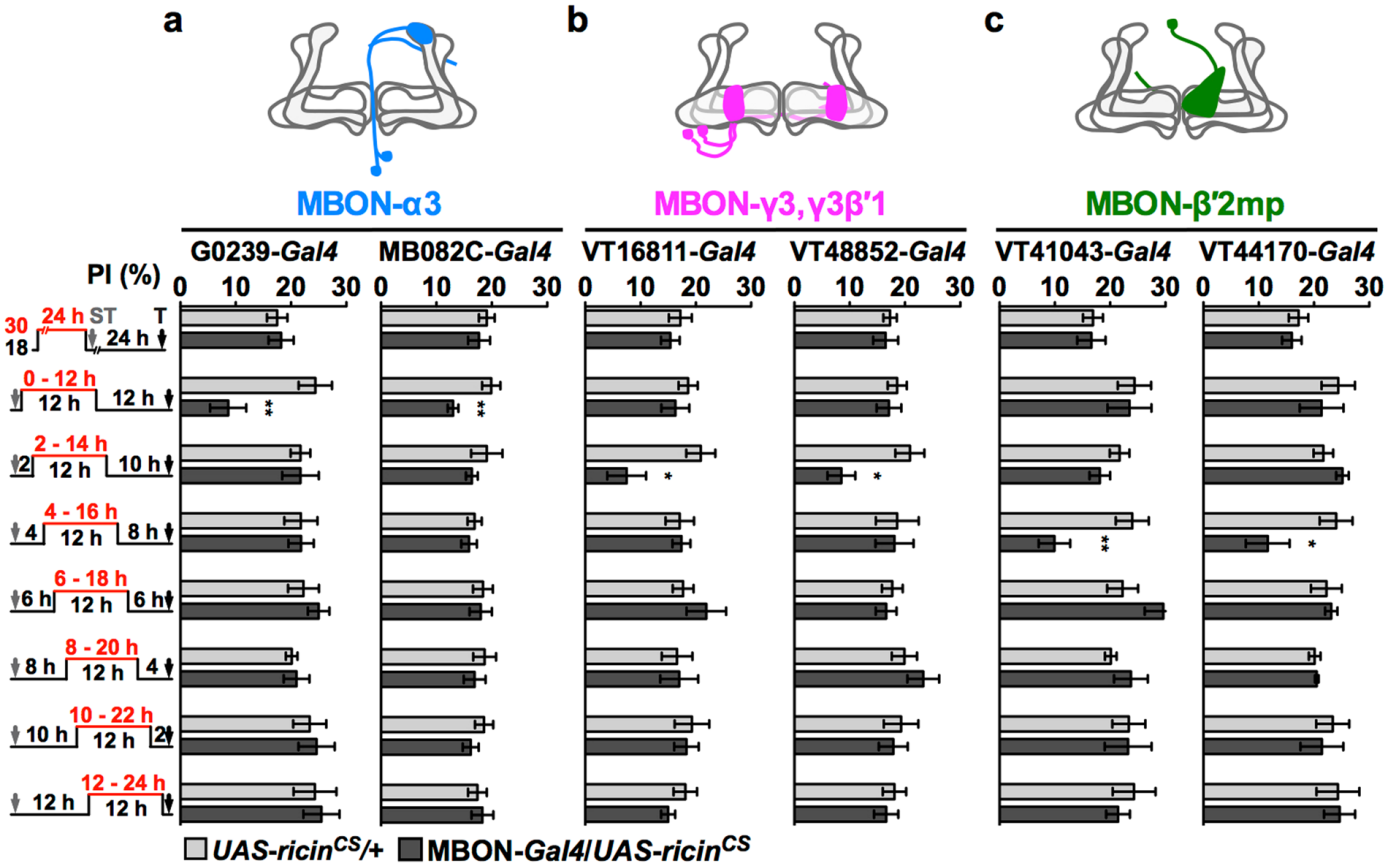
Sequential requirements of new proteins in MBON-α3 (blue), MBON-γ3,γ3β′1 (magenta), and MBON-β′2mp (green) for LTM formation. Flies were shifted to 30 °C to activate RICIN^CS^ for a 24-h window before spaced training (ST) or for a 12-h window with increasing delay after ST. All experiments were tested (T) 24 h after training. (**a**) Effects of RICIN^CS^ in MBON-α3 driven by G0239-*Gal4* and MB082C split-*Gal4*. (**b**) Effects of RICIN^CS^ in MBON-γ3,γ3β′1 driven by VT16811-*Gal4* and VT48852-*Gal4*. (**c**) Effects of RICIN^CS^ in MBON-β′2mp driven by VT41043-*Gal4* and VT44170-*Gal4*. Each value = mean ± SEM (n ≥ 8). *p < 0.05, **p < 0.01.

To investigate the period of learning-induced protein synthesis in the three different types of MBONs, we shortened the blocking period from 24-h to 12-h during LTM consolidation. The first 12-h blocking period by RICIN^CS^ was postponed by 2 h for each set of experiments with an increasing delay from immediately after spaced training (0–12 h) to before testing (12–24 h). The blocking of protein synthesis in MBON-α3, only at 0–12 h of consolidation, impaired LTM; however, LTM was still intact when protein synthesis was blocked in the six later sets (2–24 to 12–24 h) (Fig. 2a). Massed-training-induced ARM was intact after blocking 0–12 h protein synthesis in MBON-α3 (Supplementary Fig. 2a). Surprisingly, when applying the same manipulation, we found that blocking protein synthesis during 2–14 h in MBON-γ3,γ3β′1 and during 4–16 h in MBON-β′2mp after spaced training impaired LTM, but other blocking periods did not affect the LTM score (Fig. 2b,c). Blocking protein synthesis in MBON-γ3,γ3β′1 and MBON-β′2mp at the same LTM defect period was associated with an intact ARM (Supplementary Fig. 2b,c).

These results obtained from shifting the blocking period to increasingly delayed time points indicated that protein synthesis for LTM formation in these three types of MBONs was required at a specific starting point, and the order of protein synthesis was MBON-α3, MBON-γ3,γ3β′1, and MBON-β′2mp. Next, we investigated the minimum blocking period to impair LTM in these three types of MBONs. We further shortened the blocking period to 10 h or 8 h, but the starting point of the period was kept the same as in previous LTM defect conditions for each of the three types of MBONs. A period of neither 10-h nor 8-h blocking decreased the LTM score in the three types of MBONs (Supplementary Fig. 2d–f). Furthermore, 24-h blockage of protein synthesis before spaced training did not affect the LTM (Fig. 2, first row), indicating that neuronal physiology or memory acquisition of the three types of MBONs was not affected by 24-h active RICIN^CS^. These results showed that spaced training triggers the new protein synthesis required for LTM formation in specific MBONs at precise time points lasting for a certain period.

Next, we simultaneously blocked protein synthesis in the three types of MBONs. Our results showed the same degree of LTM impairment after spaced training (Supplementary Fig. 3a). ARM after massed training remained intact (Supplementary Fig. 3b). Permissive temperature control showed normal LTM after spaced training (Supplementary Fig. 3c). These findings suggested that each of these three types of MBON is individually necessary for LTM formation.

### All three types of MBONs use ORB proteins to support LTM formation

**Figure 3:**
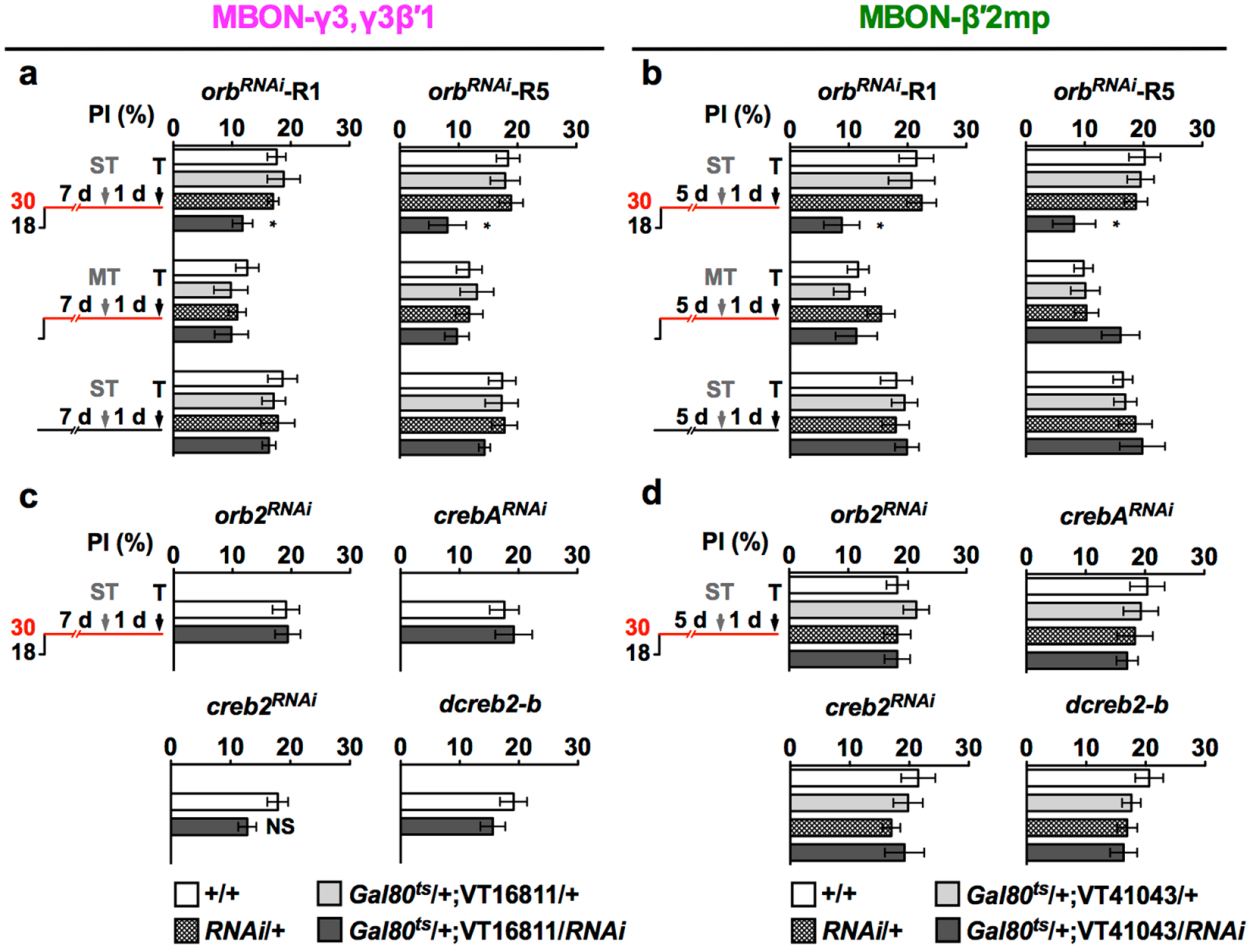
The effects of RNAi-mediated downregulation of protein synthesis regulators on LTM. Flies were trained (ST: spaced training; MT: massed training) and then tested (T) following adult-stage-specific *UAS*-RNAi induction in MBON-γ3,γ3β′1 for 7 days or in MBON-β′2mp for 5 days (30 °C). In both types of MBONs, downregulation of either *orb* ^*RNAi*^-10868R-1 (R1) or *orb* ^*RNAi*^-10868R-5 (R5) impaired 24-h LTM after ST, but not after MT (**a**,**b**). In contrast, *orb2* ^*RNAi*^-, *creb2* ^*RNAi*^-, *dcreb2* blocker (*dcreb2*-*b*)-, and *crebA* ^*RNAi*^-mediated downregulation did not affect LTM (**c**,**d**). In control experiments on flies carrying the same transgenes kept at 18 °C, or with no effector (*tub*-*Gal80* ^*ts*^/+;VT-*Gal4*/+) and effector alone (*UAS*-RNAi/+) kept at 30 °C, LTM was also not affected. Each value = mean ± SEM (n ≥ 8). Not significant (NS): p > 0.05; *p < 0.05.

Abundant evidence indicates that LTM requires the consolidation of synaptic plasticity by new learning-induced proteins through CREB-dependent transcription and/or ORB-regulated translation in the neural assembly encoding memory information^20–24^. Thus, we investigated whether these regulatory proteins are also involved in protein-synthesis-dependent LTM formation in MBON-γ3,γ3β′1 and MBON-β′2mp. We performed adult-stage-specific downregulation of target proteins in MBONs under the control of temperature-sensitive *tub*-*Gal80* ^*ts*^ to avoid possible abnormal development. As determined by monitoring GFP expression, the *tub*-*Gal80* ^*ts*^ induction times for MBON-β′2mp and MBON-γ3,γ3β′1 were 5 and 7 days, respectively (Supplementary Fig. 4a,b)^41^. For the given induction time in MBON-γ3,γ3β′1 or MBON-β′2mp, neither RNAi-mediated downregulation of *crebA* and *creb2* genes nor the overexpression of the *dcreb2* blocker (*dcreb2*-*b*) affected LTM after spaced training (Fig. 3c,d). In contrast, downregulating *orb* by two independent lines of RNAi in MBON-γ3,γ3β′1 or MBON-β′2mp impaired LTM (Fig. 3a,b), but RNAi-mediated *orb2* downregulation had no effect (Fig. 3c,d). Massed training control showed normal ARM after RNAi-mediated *orb* downregulation (Fig. 3a,b, MT), suggesting that ORB proteins in MBON-γ3,γ3β′1 and MBON-β′2mp are involved only in protein-synthesis-dependent LTM formation. An uninduced *tub*-*Gal80* ^*ts*^ control at 18 °C did not affect LTM, indicating the specificity of the RNAi and blocker (Fig. 3a,b, non-heat-shock ST). We further confirmed the requirement of ORB in MBON-γ3,γ3β′1 and MBON-β′2mp for LTM with an independent driver for each MBON (Supplementary Fig. 4c,d). Adult-stage-specific ORB downregulation in MBON-γ3,γ3β′1 and MBON-β′2mp did not affect learning (Supplementary Fig. 4e,f), indicating that these flies exhibited normal CS/US association. Together with a previous report^22^, our results showed that all three types of MBONs (i.e., MBON-α3, MBON-γ3,γ3β′1, and MBON-β′2mp) require ORB, a local translational *cpeb* mRNA binding protein, to form aversive olfactory LTM.

### MBON-γ3,γ3β′1 and MBON-β′2mp are parts of the LTM retrieval circuit

**Figure 4:**
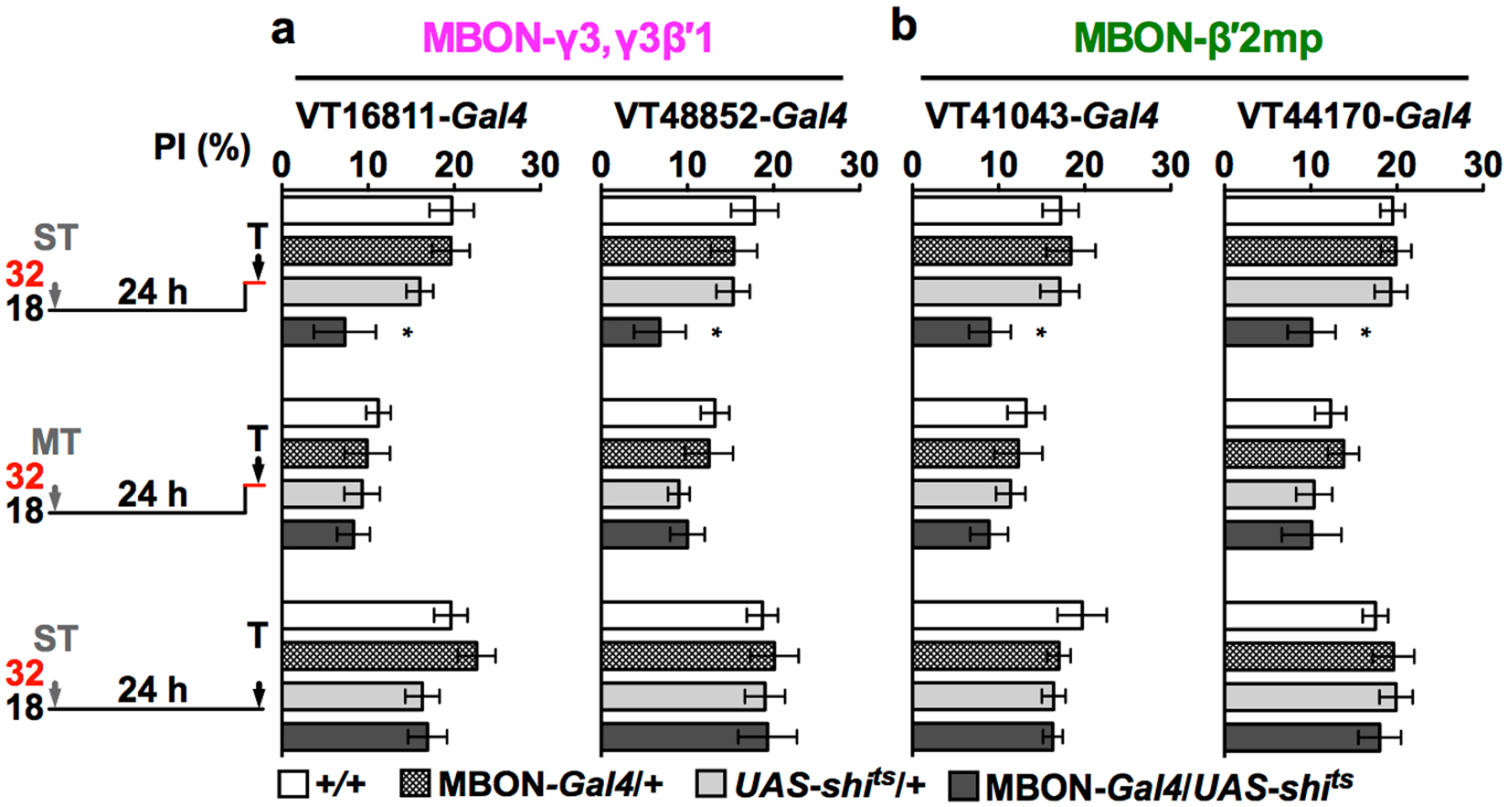
Blocking neurotransmission outputs of MBON-γ3,γ3β′1 or MBON-β′2mp during memory retrieval impaired LTM. Flies were trained with spaced training (ST) or massed training (MT) and were then shifted to a restrictive temperature (32 °C) 40 min before the test to block neurotransmission output during testing (T). The effects of activated SHI^ts^ during LTM retrieval in MBON-γ3,γ3β′1 (**a**) or MBON-β′2mp (**b**). Inactivating neurotransmission output during retrieval after MT did not affect ARM in MBON-γ3,γ3β′1 or MBON-β′2mp. Driver alone (MBON-*Gal4*/+) and effector alone (*UAS*-*shi* ^*ts*^) controls showed normal LTM under the same treatment, and permissive temperature controls for all genotypes had normal LTM. Each value = mean ± SEM (n ≥ 8). *p < 0.05.

The recall of protein-synthesis-dependent LTM requires the reactivation of memory-encoding neurons to retrieve memory contexts^7, 8, 42^. As MBON-α3 outputs are required during LTM retrieval^22^, we investigated whether this is also the case in MBON-γ3,γ3β′1 and MBON-β′2mp by acutely blocking dynamin-mediated endocytosis for recycling of neurotransmitter and receptor vesicles with a temperature-switchable mutant *UAS*-*shi* ^*ts*^ ^43, 44^. LTM retrieval was impaired in flies with inactivated output of MBON-γ3,γ3β′1 or MBON-β′2mp at a restrictive temperature (32 °C) (Fig. 4a,b, heat-shock ST). In contrast, ARM retrieval was normal in both cases after the same manipulation (Fig. 4a,b, heat-shock MT). We confirmed these results with independent *Gal4* drivers (Fig. 4, VT48852 and VT44170) and MBON-specific split-*Gal4* lines (Supplementary Fig. 5). In addition, inactivating these two neurons during training still resulted in intact LTM (Supplementary Fig. 6a,b), and 1X learning was also intact when output was inactivated throughout training and testing (Supplementary Fig. 6c,d). Taking these findings into account together with the RICIN^CS^ result, we concluded that the three types of MBONs (i.e., MBON-α3, MBON-γ3,γ3β′1, and MBON-β′2mp) became parts of the LTM retrieval circuit by learning-induced protein synthesis, without affecting ARM formation or retrieval^22^. Furthermore, the *shi* ^*ts*^ results implicated that neurotransmissions from these three neurons are necessary for the recall of LTM.

### MBON-α3 and MBON-β′2mp connect to the DAL neurons

**Figure 5:**
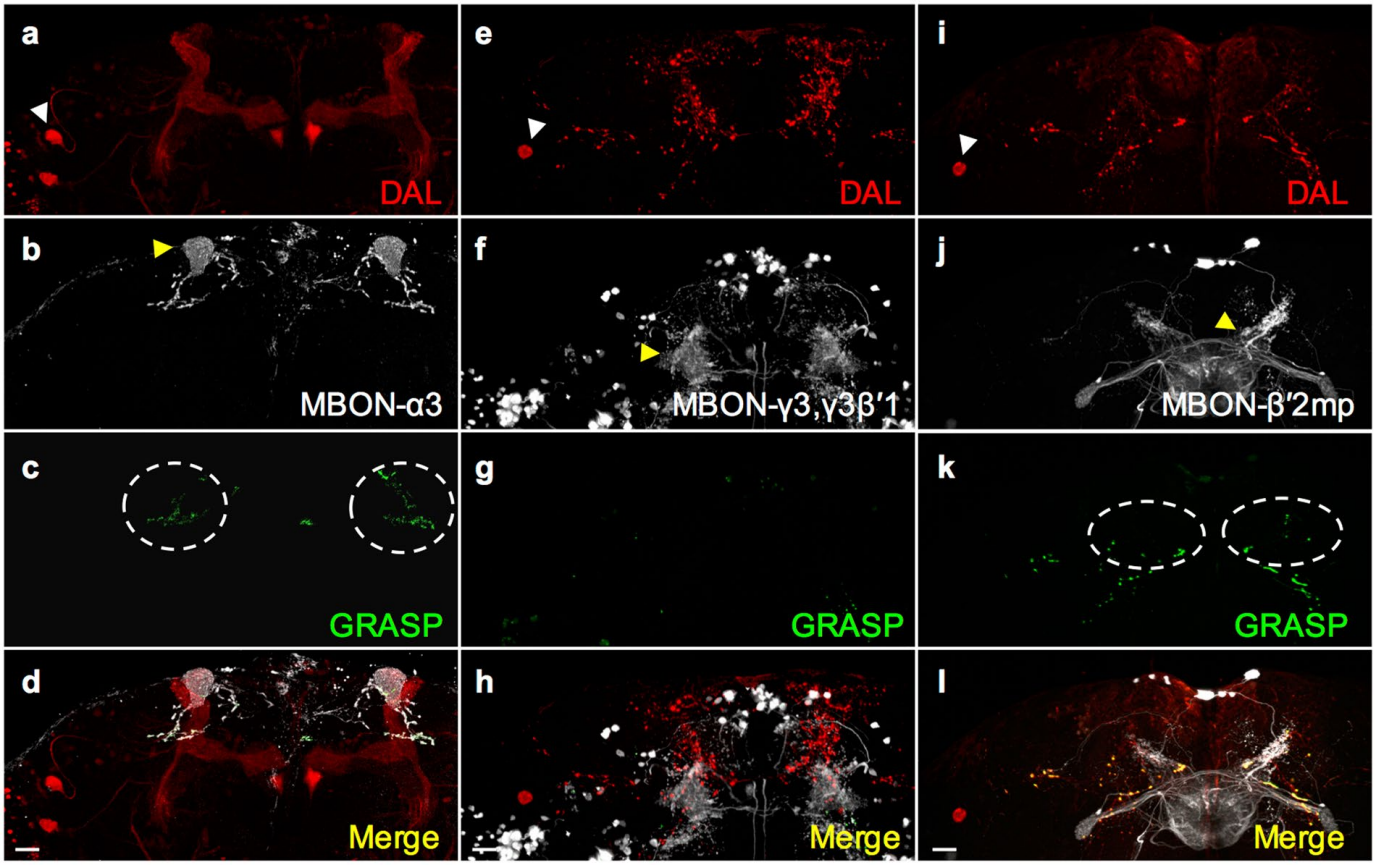
DAL as the downstream neurons of MBON-α3 and MBON-β′2mp. The connectivity of MBON-α3/MBON-β′2mp and DAL was visualized by GRASP. DAL (**a**,**e**,**i**) and MBONs (**b**,**f**,**j**) contributed GRASP signal in (**c** and **k**). The white dashed circle indicates the connectivity of DAL and MBONs (**c** in SMP and SIP; **k** in CRE). The white arrowhead indicates soma of DAL, and the yellow arrowhead indicates the innervated compartments of MBON. *LexA* drivers were visualized by *LexAOP*-mKO (red), and *Gal4* drivers were visualized by anti-GFP immunostaining (gray). Scale bar for all images: 50 µm.

The three types of MBONs have all been shown to project axons to the dorsal brain regions, including the crepine (CRE), superior medial protocerebrum (SMP), superior intermediate protocerebrum (SIP), and/or superior lateral protocerebrum (SLP) (Supplementary Fig. 7a–h)^22, 28, 41^. LTM retrieval also requires neurotransmission from the DAL neurons that project dendrites into CRE, SMP, and SIP^21^. To understand LTM information processing at the system level, we applied GFP reconstitution across synaptic partner (GRASP)^45^ to check whether these three types of MBONs connect anatomically to the DAL neurons. We expressed the two split-GFP partners spGFP_1–10_ and spGFP_11_ in all three types of MBONs and the DAL neurons, respectively. In flies carrying DAL-*LexA* > *LexAOP*-spGFP_11_ (Fig. 5a) and MBON-α3-*Gal4* > *UAS*-spGFP_1–10_ (Fig. 5b), we observed GRASP signals in the SMP and SIP regions (Fig. 5c). In flies carrying DAL-*Gal4* > *UAS*-spGFP_1–10_ (Fig. 5e) and MBON-γ3,γ3β′1-*LexA* > *LexAOP*-spGFP_11_ (Fig. 5f), we did not see any GRASP signals (Fig. 5g). In flies carrying DAL-*Gal4* > *UAS*-spGFP_1–10_ (Fig. 5i) and MBON-β′2mp-*LexA* > *LexAOP*-spGFP_11_ (Fig. 5j), we observed GRASP signals in the CRE region (Fig. 5k). These findings suggested the existence of two LTM consolidation and retrieval circuits converging at the DAL neurons: MB → MBON-α3 → DAL and MB → MBON-β′2mp → DAL. The downstream neurons of MBON-γ3,γ3β′1 remained unknown.

## Discussion

At the cellular level, we found that MBON-γ3,γ3β′1 and MBON-β′2mp underwent new protein synthesis to consolidate LTM, in addition to the previously identified MBON-α3 (Fig. 1)^22^. All these three types of MBONs required the ORB to form LTM (Fig. 3)^22^. At a specific synapse, neuronal stimulus can induce the phosphorylation of the ORB, which activates local mRNA translation by working with poly(A) binding proteins to form new proteins^6, 14^. Here, our data support a LTM formation model in which the ORB activated by spaced training triggers synapse-specific local mRNA translation and consolidates CS/US coding from sparse KCs to specific postsynapses of the three types of MBONs^31, 35, 36^. This local ORB activation sequentially triggered new protein synthesis that persisted for at least 12 h in MBON-α3, then -γ3,γ3β′1, and finally -β′2mp to form LTM (Fig. 2; Supplementary Fig. 2d–f). These ORB proteins were probably already present at postsynapses rather than newly synthesized since they were immediately required after spaced training in MBON-α3 (Fig. 2a). Consistent with this, spaced association increases the levels of several mRNAs in MBON-α3, without significantly altering the level of the *orb* mRNA^46^. It is important to determine whether downregulating the ORB abolishes mRNA elevation by spaced association.

At the system level, these three types of MBONs were triggered sequentially to synthesize new protein during LTM consolidation (Fig. 2) and demanded neurotransmission output during LTM retrieval (Fig. 4)^22^. Interestingly, their outputs were not required during CS/US information acquisition for learning as well as LTM (Supplementary Fig. 6). Previous rescue experiments demonstrated that D1-like dopamine receptor-mediated US is only required in γ KCs, for all stages of memory formation, including learning and LTM^47^. This study and several others also demonstrated a requirement for outputs from specific α/β and α′/β′ KCs during LTM consolidation and retrieval^48–50^ and for protein synthesis in MBONs from various MB lobes for LTM formation. The perplexing findings may have stemmed from the presence convoluted circuits, as axons of MBONs overlap with dendrites of dopaminergic MBINs to form recurrent loops, which are involved in both aversive and appetitive memory formation^28, 35, 36, 51^. In this study, the GRASP data suggest another possible MB recurrent loop in which DAL neurons connect MBON-α3 and MBON-β′2mp (Fig. 5c,k) to the calyx K5 region of pioneer α/β subset^21^. Because LTM formation requires learning-induced new protein synthesis and functional NMDA receptors in the DAL neurons, we speculate that the glutamatergic MBON-β′2mp and the cholinergic MBON-α3 may act together to activate the DAL^21, 28, 34, 52^. Moreover, the recurrent connections to pioneer α/β may allow DAL to modulate MB activities that trigger sequential protein synthesis in the MBONs (Fig. 2). These results raise the possibility that these “MB loops” acting as memory-encoding circuits can sustain learning-induced neural activity and trigger new protein synthesis in the DAL neurons and the three types of MBONs in order to consolidate long-lasting memory. When a fly needs to recall a memory, CS reactivates these LTM storage neurons in the MB loops and recruits additional neurons in the retrieval circuits (such as MBON-V2 cluster, ellipsoid body neurons, and K5 KCs) to anticipate US without an actual stimulus^21, 33, 53, 54^. This recurrent-loop model also predicts a requirement of outputs from DAL and MBONs during LTM consolidation. In addition, LTM retrieval likely involves other neurons since the MBON-γ3,γ3β′1 downstream neurons remain unknown and axonal terminals of MBON-α3 and MBON-β′2mp are widely distributed (Fig. 5)^22, 28, 41^. Notably, putative contacts indicated by positive GRASP signals still need to be verified to determine whether they form functional synapses by monitoring DAL responses to the activation of specific MBONs.

The mouse amygdala, as a hub at which multiple sensory inputs converge to associate with experiences, encodes positive or negative valence at the output level^55^. Fly MBONs also have an intrinsic valence, including neutral, approach, or avoidance, when triggered individually by optogenetics^34, 36^. We speculated that each of the three types of MBONs plays a distinct role in LTM formation^32^. The cholinergic MBON-α3 exhibits a neutral valence, indicating that flies neither approach nor avoid the optogenetic activation^34^. By 10X optogenetic activation of the dopaminergic PPL1-α3 upstream of MBON-α3 paired with an odor (CS+), flies form LTM lasting for at least 4 days^32^. These results suggest a critical long-lasting US-encoding circuit at the α lobe tip: PPL1-α3 → KC(α3) → MBON-α3. This would explain why new protein synthesis was required first after training in MBON-α3 (Fig. 2a) and why the α lobe was critical for appetitive and aversive LTM formation^22, 33, 34, 49, 56, 57^. The second protein-synthesis-dependent GABAergic MBON-γ3,γ3β′1 encodes a strong approach valence, while the activation of its dopaminergic PAM-γ3 input substitutes sufficiently for the US signal during aversive learning^34, 58^. A possible explanation of this conceptual disagreement is that the activation of PAM-γ3 → MBON-γ3,γ3β′1 circuits drives the approach toward CS- (the odor paired without shock) as a “relief learning” cue^59–61^. The third protein-synthesis-dependent MBON-β′2mp encodes an avoidance valence involved in both innate and learned behaviors, including cold avoidance, CO_2_ avoidance, reward memory, visual memory, and intermediate-term memory^34, 41, 62–66^. MBON-β′2mp exhibits an increased calcium response to CS+ after aversive conditioning and, as mentioned previously, MBON-β′2mp anatomically forms a loop with its upstream MBINs, which might perpetuate information to strengthen avoidance valence^36^. Furthermore, the early activity from α′/β′ KCs after aversive training is required for intermediate-term memory^67, 68^, suggesting that the perpetuation of neuronal activity in this loop maintained the activity flow and triggered protein synthesis of MBON-β′2mp to consolidate aversive LTM contexts for avoidance behavior (Fig. 2c). Combining with the multiple functions of MBON-β′2mp, we speculated that the MB β′2 compartment module serves as a high-weighting avoidance valence center when animals need to perform an escaping behavior. Interestingly, neurotransmission outputs from the three types of MBONs were all required during LTM retrieval (Fig. 4), suggesting that each represents partial LTM contexts and all contexts are necessary for LTM performance (Supplementary Fig. 3). Although how the valences of the three types of MBONs were affected by new learning-induced proteins remains unclear, the combination of distinct MB modules (MBIN-KC-MBON) provides a flexible circuitry array that processes and stores distinct experiences so that appropriate behaviors can be enacted when encountering the same situations again^34, 69^.

The three types of MBONs in the *Drosophila* brain fulfill the following three criteria to match the definition of a “memory engram neuron” discussed widely in rodents^7, 8, 42, 70^: (*i*) training-induced neuronal activation, (*ii*) training-induced chemical and/or physical changes, and (*iii*) recall-induced reactivation. All MBONs respond to odors^71^. Their responses, especially those of MBON-α3 and -β′2mp, to a CS are modulated by dopaminergic MBINs pairing with a US [fulfilling (*i*)]^22, 31, 35, 36^. Our results of protein synthesis blocking and neurotransmission inactivation revealed that the synthesis of new proteins modified LTM plasticity in the three types of MBONs, and then the activation of this modified plasticity was required at the later memory recall (Figs 2 and 4) [fulfilling (*ii*) and (*iii*)]. Thus, our work shows that the three types of MBONs, -α3, -γ3,γ3β′1, and -β′2mp, serve as LTM engram neurons at the output level of the MB and reveals the strategy that they coordinate systematically to encode LTM in the *Drosophila* brain.

## Methods

### Fly strains

Fly lines were kept on standard corn meal/yeast/agar medium at 25 ± 0.5 °C (for imaging) or 18 ± 0.5 °C (for behavior assay) and 70% relative humidity under a 12-h:12-h light:dark cycle. The fly lines used were wild-type Canton-S w1118 (iso1CJ), and all fly lines used for behavior assays were outcrossed to the wild type for at least five generations.

VT lines were provided by B.J. Dickson (VDRC) and were used as MBON drivers to perform all experiments. MBON split-*Gal4* drivers were used as more specific independent drivers to prove the behavior outcome^28^. G0239-*Gal4* for MBON-α3 was from Bloomington. *UAS*-*shi* ^*ts*^ was used to block neurotransmission. For downregulating gene expression, *orb* RNAi-R1, *orb* RNAi-R5 (National Institute of Genetics, Shizuoka), *orb2* RNAi (K. Si), *crebA* RNAi (V108357, VDRG), *creb2* RNAi (v101512, VDRC), and *dcreb2* blocker (R.L. Davis) were expressed in adults under *tub*-*Gal80* ^*ts*^ (Bloomington) control at 30 ± 0.5 °C for 5 days in MBON-β′2mp drivers^41^, but in MBON-γ3,γ3β′1 drivers, RNAi was expressed for 7 days. *UAS*-mCD8::GFP;*UAS*-mCD8::GFP and *LexAOP*-mKO were used to visualize driver expression for *Gal4* and *LexA*, respectively. *UAS*-Dscam::GFP;*UAS*-mKO,*UAS*-mKO;*UAS*-Syt::HA was used for a survey of polarity^41^. GRASP, *UAS*-CD4::spGFP_1–10_, and *LexAop*-CD4::spGFP_11_ were used to verify connectivity between two neurons driven by *Gal4* and *LexA* (K. Scott). L0124-*LexA* labeled whole KCs as an MB landmark.

### Generation of transgenic lines

The transgenic flies VT41043-*LexA* (MBON-β′2mp), VT16811-*LexA* (MBON-γ3,γ3β′1), and VT49239-*LexA* (DAL) were constructed as described based on *Gal4* line expression patterns from our laboratory. The PCR products were amplified by using the *Drosophila* genome as a template and specific primers based on promoter fragment sequences of VT41043, VT16811, and VT49239 from VDRC. These PCR products were sequenced and cloned into the pPBLexA::p65Uw vector (Addgene, #26231), which contained an attB site-specific recombination region. Then, the transgenic flies were obtained by phiC31-integrase-mediated transgenesis with insertion in attP2.

### RICIN^CS^ specificity

The temperature-dependent ribosomal toxin RICIN^CS^ was originally designed for the ablation of *Drosophila* eye cells^39^. By remobilization of the original *UAS*-*ricin* ^*CS*^ P-element transposon, we previously obtained two effective *UAS*-*ricin* ^*CS*^ lines for blocking new protein synthesis within a day without killing the target cells^21^. Briefly, using a photoconvertible KAEDE fluorescence protein as a protein synthesis reporter, we show that the effective RICIN^CS^ can block new KAEDE synthesis in several different target cells for at least 24 h at 30 °C. This is not due to killing the cells because KAEDE synthesis resumes when flies return to 18 °C. The RICIN^CS^ specificity for blocking protein synthesis is also indicated by a behavior assay showing that 24-h RICIN^CS^ activation in all brain neurons is functionally equivalent to cycloheximide feeding in suppressing LTM formation. In addition, RICIN^CS^ inhibition together with cycloheximide feeding does not further suppress LTM^21^. In this study, we show that LTM is impaired by activating RICIN^CS^ in specific MBONs after, but not before, spaced training for the same duration (Figs 1 and 2), suggesting that the target cells survive and function normally when flies return to 18 °C after 24 h at 30 °C.

### Behavioral assays

Olfactory aversive learning was performed by pre-dried 2- to 5-day-old flies with a Pavlovian olfactory conditioning procedure^25^. Approximately 100 flies received one or ten sessions of spaced or massed training, in which the flies were exposed sequentially to one odor (CS+) paired with an electrical foot shock for 60 s and then to 45 s of normal air following by a second odor (CS−) without a shock for 60 s. The two odors used were 3-octanol (218405, Sigma-Aldrich) and 4-methylcyclohexanol (153095, Sigma-Aldrich), which were diluted in mineral oil (330760, Sigma-Aldrich) at ratios of 1.5:1000 and 1:1000, respectively. For 24-h memory, an automated robot-trainer was used for training with spaced or massed training w/o a 15-min interval and the trained flies were then transferred to regular food and incubated at 18 °C or a restrictive temperature (RICIN^CS^ at 30 ± 0.5 °C and SHI^ts^ at 32 ± 0.5 °C) and 70% relative humidity in a dark chamber. The trained flies were then shifted to a testing room 40 min before the test to acclimate to the testing conditions and then tested with a T-maze apparatus for 2 min. All genotypes were trained and tested in parallel and rotated among eight robot-trainers to ensure a balanced experiment. Learning was performed using the T-maze in the whole training and testing protocol. A performance index was calculated as the number of flies avoiding the CS+ minus those avoiding the CS−, divided by the total number of flies and finally multiplied by 100; the obtained values were then averaged for two reciprocal experiments to reduce the bias between the two odor preferences, non-associative changes in olfaction, or the particular tube within the T-maze that the flies chose to enter. All LTM defect experiments were repeated by the experimenter blinded to the genotype.

### Whole-mount immunostaining and imaging

An adult fly brain was prepared and imaged as previously described^21^. The dissected brain was incubated with primary antibody at 4 °C for two days, and the dilution ratios were 1:10 for mouse 4F3 anti-DLG (Hybridoma Bank, University of Iowa), 1:500 for rabbit anti-HA (Abcam), and 1:500 for rabbit anti-GFP (Invitrogen). The secondary antibody solution with the fly brain was incubated at room temperature overnight, and the dilution ratio was 1:200 for biotin-conjugated goat anti-mouse IgG (Invitrogen) and biotin-conjugated goat anti-rabbit IgG (Invitrogen), and then the fly brain was labeled by 1:200 dilution with Alexa Fluor 635 streptavidin (Invitrogen) at room temperature overnight.

### Statistics

All of the behavioral raw data were analyzed parametrically with JMP^®^ 9.0.0 software. Owing to the nature of their mathematical derivation, performance indexes were distributed normally. Hence, data were evaluated via one-way ANOVA. Subsequent pair-wise planned comparisons were adjusted for experiment-wise error (α′), keeping the overall α = 0.05. All data are presented as mean ± SEM.

## Electronic supplementary material

Supplementary Infromation
