# Supplementary material for: Long-term memory requires sequential protein synthesis in three subsets of mushroom body output neurons in Drosophila
Source: Sci Rep. 2017 Aug 2;7:7112. doi: 10.1038/s41598-017-07600-2 (PMC5540930; doi:10.1038/s41598-017-07600-2)
Supplement: Supplementary file 1 — Supplementary Infromation [file 41598_2017_7600_MOESM1_ESM.pdf]

Supplementary information

Long-term memory requires sequential protein synthesis in three subsets of mushroom body output neurons in *Drosophila*

Jie-Kai Wu[1], Chu-Yi Tai[1], Kuan-Lin Feng[1], Shiu-Ling Chen[2], Chun-Chao Chen[2], and Ann-Shyn Chiang[1,2,3,4]*

[1]Institute of Biotechnology, National Tsing Hua University, Hsinchu 30013, Taiwan

[2]Brain Research Center, National Tsing Hua University, Hsinchu 30013, Taiwan

[3]Genomics Research Center, Academia Sinica, Nankang, Taipei 11529, Taiwan

[4]Kavli Institute for Brain and Mind, University of California, San Diego, La Jolla, CA 92093-0526, USA

Correspondence and requests for materials should be addressed to A.-S.C. (email: aschiang@life.nthu.edu.tw)

Expression in the MB compartments[28]

Lines	γ					α'β'					αβ					C
						α'			β'		α			β		
	1	2	3	4	5	1	2	3	1	2	1	2	3	1	2	
E0328										+						
E0330		++														
E0384		++			+						+	+		+	+	
E0506															+	
E0565#																
E0916#																
E0925#																
E1130	+															
E1132**							+						+			
E1255					++											
E1290#																
E1472															+	
E1473**															+	
G0239***													+			
G0516															+	
VT00351	++	++		+		++							+			
VT00765															++	
VT01611**			+						+							
VT02081																+
VT02214				++												
VT02216		+				+	++					++				
VT02472																
VT04425	+	+	+	+	+	+	+	+	+	+	+	+	+	+	+	
VT04977	+	+	++	++					++					++		
VT04983*	++															
VT04995	+	+	+	+	+	+	+	+	+	+	+	+	+	+	+	+
VT05010	+		++						++							
VT05526			+		+				+							
VT05948											+			++		
VT06202					+					+					+	
VT06537					++					++						
VT06555			+	++	++				++	++					+	
VT07174										++					+	
VT07740**				++										++		
VT07759*										++					+	+
VT08145															++	
VT08154**			++							+						
VT08167									++							
VT08645																+
VT08669*						+	+									
VT08685	+	+	+		++											
VT08886					+										++	
VT08967	+	+		++												
VT09573				+							+					
VT10033700																
VT10260	++					+										
VT11035***					++					++						+
VT11128*	+	+	+	+	+											
VT12717				+						++						
VT12761	++					+										
VT13944																+
VT14712					++											
VT16276**														+	+	
VT16465	++															
VT16650											+					+
VT16671												+			+	
VT16811*			++						++							
VT17126					++					++					+	
VT17260		+	+		+				+	+	+	+	+	+	+	
VT19028							+					++				++
VT19257																+

Expression in the MB compartments[28]

Lines	γ					α'β'					αβ					C
						α'			β'		α			β		
	1	2	3	4	5	1	2	3	1	2	1	2	3	1	2	
VT19433	++										++					
VT19739		+									+					
VT19753			+		++					+					+	
VT19841	+	+	+	+	+				++	++	++			++		
VT19842				++					+	++	++			+	+	
VT19843	++		++						++							+
VT19992																+
VT20611												+				
VT23472	++				+					++						
VT23820										++						
VT24617	+										++					
VT24618	++										++					
VT25391***		++				++								++		
VT25604	+	+	+	+	+	+	+	+	++	+	+	+	+	+	+	
VT25781															++	
VT25996				+	+											
VT25999							+									
VT26013	+									++						
VT26018										+						
VT26020																+
VT26023	+			++	++					+						
VT26183		+				+								+		
VT26341																++
VT26347*	+	+	+						+		+					+
VT26659	++															
VT27004								+				+				
VT28150	+			++	++					+						
VT29590				+												
VT30286											+			+		
VT30599**										+						
VT32110	++	++		+	+	++				++						++
VT34816																++
VT36875									++							
VT37873										++						
VT38211*									+							+
VT38795				++										++		
VT38815	+	+		++												+
VT39760*	+					+										++
VT39763											+			+	+	
VT40010	++				+					+		+				++
VT40022				+	+											
VT40045										+						
VT40350					++					++	+					
VT40924										++						
VT40925										++						
VT41043***										++						
VT41878												++				
VT43014										+						
VT43068	+			+										+		+
VT43140	+			+										+		
VT43156					++					+					++	
VT43326	+			+	++					++	+				++	
VT43361										+				+		
VT43401												+				
VT43692	+	+		+	+											
VT44170**										++						
VT44322										+	++					
VT44326#																
VT44648				+												++
VT44833	++															
VT44958	+	+		++												

Table 1. continued on next page

Table 1. continued

Lines	Expression in the MB compartments[28]															
	γ					α'β'					αβ					C
						α'			β'		α			β		
	1	2	3	4	5	1	2	3	1	2	1	2	3	1	2	
VT45508***							+						+			
VT45584*					++					+						
VT45654			++						++							
VT45661**	++	+														
VT45971					++	+									++	
VT45997	+															
VT45998																++
VT45999						+	+									
VT46082		++				++	++									
VT46265																+
VT46525					+											
VT46792										++						
VT46805									++							
VT46826	+	+		+	+	+				++					++	++
VT47323	+	+	+	+	+	+	+	+	+	+	+	+	+	+	+	+
VT48029		+				+										+
VT48153					++					++						
VT48340										+		+				
VT48344	++															
VT48348					+											
VT48637	+	+	+	+	+					+	+					
VT48823			+						+							
VT48852*			++						++							
VT49105#																
VT49130***	++												++			
VT49135			+						+							
VT49356					+									+	+	
VT50187		++				++										
VT50240					+											
VT50245		++				++						+				
VT50658		+				+										

Lines	Expression in the MB compartments[28]															
	γ					α'β'					αβ					C
						α'			β'		α			β		
	1	2	3	4	5	1	2	3	1	2	1	2	3	1	2	
VT50733	++	+														
VT50737			++						++							
VT55419																+
VT56347#																
VT56374															+	
VT56510																++
VT57138														+	+	
VT57242					++			++								
VT57467				+												+
VT58427**																+
VT58430	+	+	+	+	+											+
VT58437					+										++	+
VT58464											+	+		+	+	
VT58708	+										+			+		
VT58719			+						+							
VT58722					++											
VT58734		+				+										
VT58738										+						
VT59784														++		
VT59883		+		+							+	++		+		
VT61717				++	++											
VT62256	+	+	+	+												+
VT63232																
VT63302					+											
VT63305																
VT63541	+	+	+	+	+	+	+	+	+	+	+	+	+	+	+	
VT63733*							++									
VT64581#																
VT65322	+															+
VT999005**																+

Table 1. A preliminary behavior screen of candidate MBONs making new proteins necessary for LTM formation. Expression patterns of MBON-*Gal4>UAS-mCD8::GFP* flies in the specific MB compartments were indicated. LTM scores of MBON-*Gal4>UAS-ricin*^{CS} flies were normalized by the score of wild-type control. Flies were raised at 18 °C, trained at room temperature, shifted to 30 °C to activate RICIN^{CS} immediately after spaced training for 24 h and then shifted back to 18 °C to inactivate RICIN^{CS} 40 min before testing. Each value = mean ± SEM ($n \geq 8$). *: $p < 0.05$, **: $p < 0.01$, ***: $p < 0.001$. Note that the screen experiments did not consider differences in genetic background and behavior response acuity. C: the calyx of MB. #: do not examine the image.

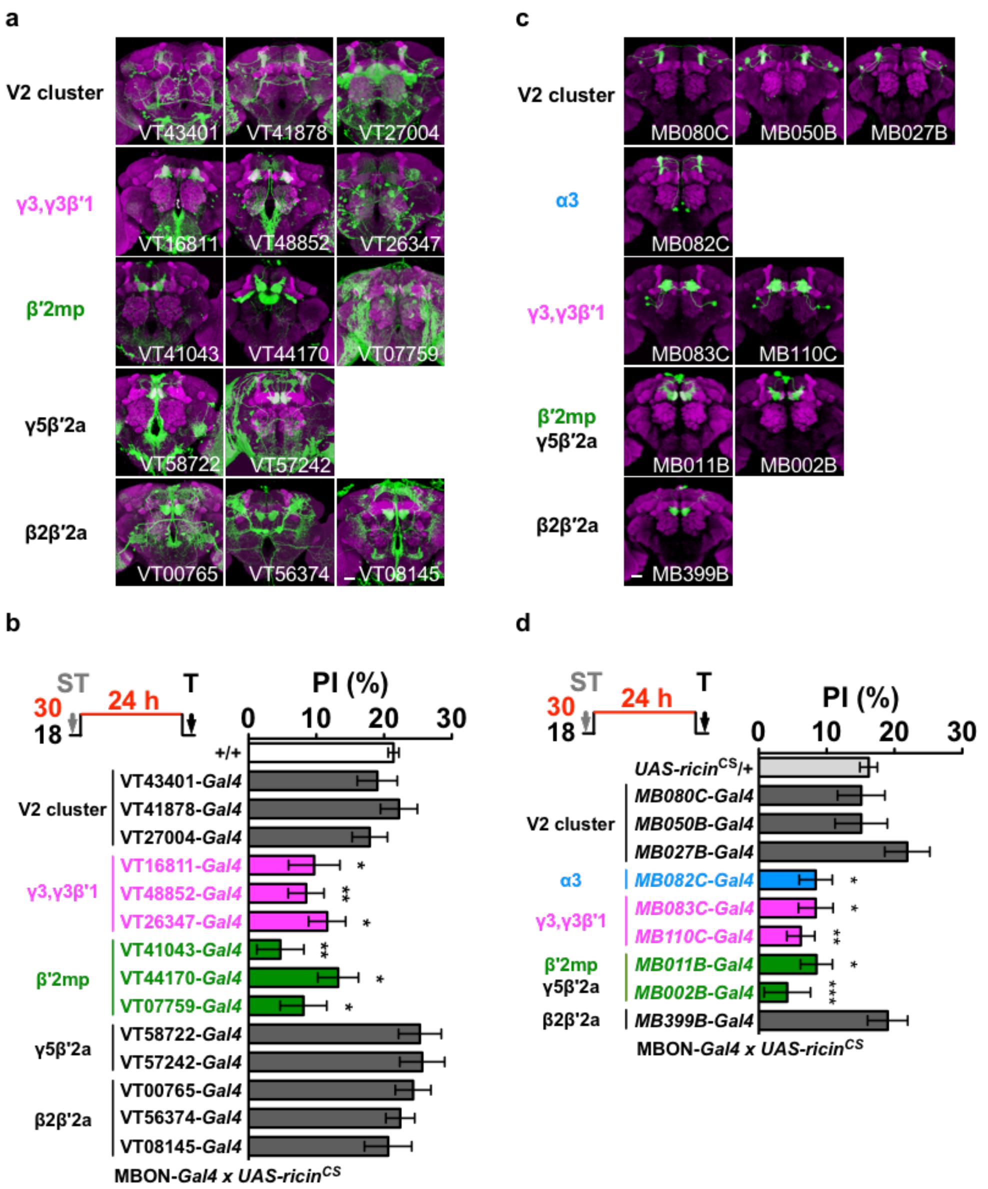

Supplementary Figure 1 | Identifying MBONs with learning-induced proteins necessary for LTM. Expression patterns of VT-*Gal4* (**a**) and split-*Gal4* (**c**) drivers visualized by *UAS*-mCD8::GFP;*UAS*-mCD8::GFP (green). Neuropils were immunostained by anti-DLG (magenta). Scale bar = 100 μm. LTM affected after RICIN^{CS} inhibition driven by *Gal4* (**b**) and split-*Gal4* (**d**) lines expressed in MBON-V2 cluster, MBON-α3 (blue), MBON-γ3,γ3β′1 (magenta), MBON-β′2mp (green), MBON-γ5β′2a, and MBON-β2β′2a. Flies were shifted to 30 °C to activate RICIN^{CS} immediately after spaced training (ST) and then shifted back to 18 °C to

inactivate RICIN^{CS} 40 min before testing (T). Each value = mean ± SEM ($n \geq 8$). *: $p < 0.05$, **: $p < 0.01$, ***: $p < 0.001$ compared with wild-type control (+/+) (white bar) or effector alone (*UAS-ricin*^{CS}/+) (light gray bar).

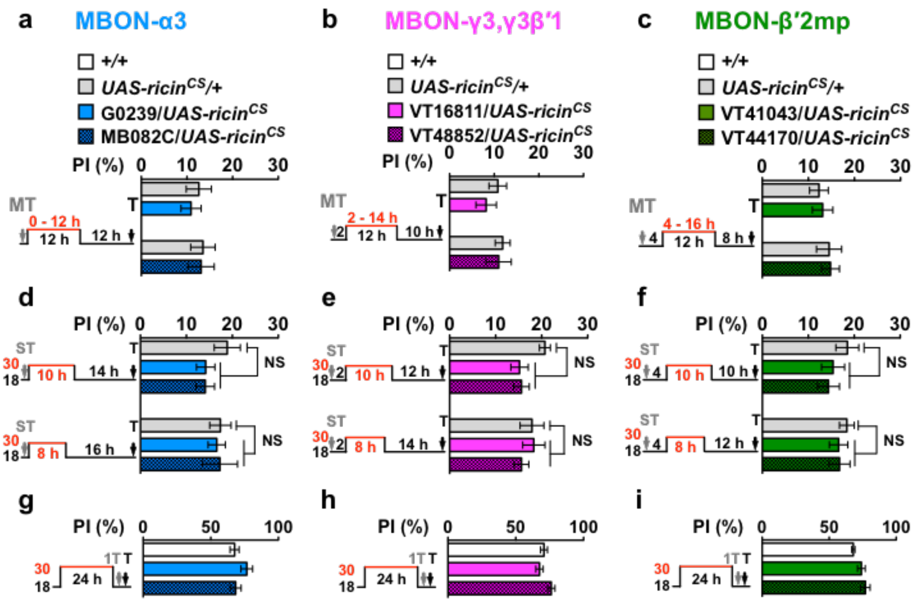

Supplementary Figure 2 | The time window of the protein synthesis requirement for LTM in the three types of MBONs. (**a–c**) Blocking protein synthesis for 12 h in the LTM-deficient periods did not affect ARM in the three types of MBONs. Flies were trained with massed training (MT) and then tested (T) for their 24-h memory. Each value = mean ± SEM ($n \geq 8$). There were no differences in ARM scores among the groups: $p > 0.05$. (**d–f**) Flies were trained with spaced training (ST) and then tested for their 24-h memory. Neither 10-h nor 8-h blocking of protein synthesis at specific time points for each affected LTM in the three types of MBONs. Each value = mean ± SEM ($n \geq 8$). Not significant (NS): $p > 0.05$. (**g–i**) Blocking protein synthesis for 24 h did not affect learning score in the three types of MBONs. Learning was performed with one session of training (1T), followed immediately by testing. Each value = mean ± SEM ($n \geq 6$). There were no differences in learning scores among the groups: $p > 0.05$.

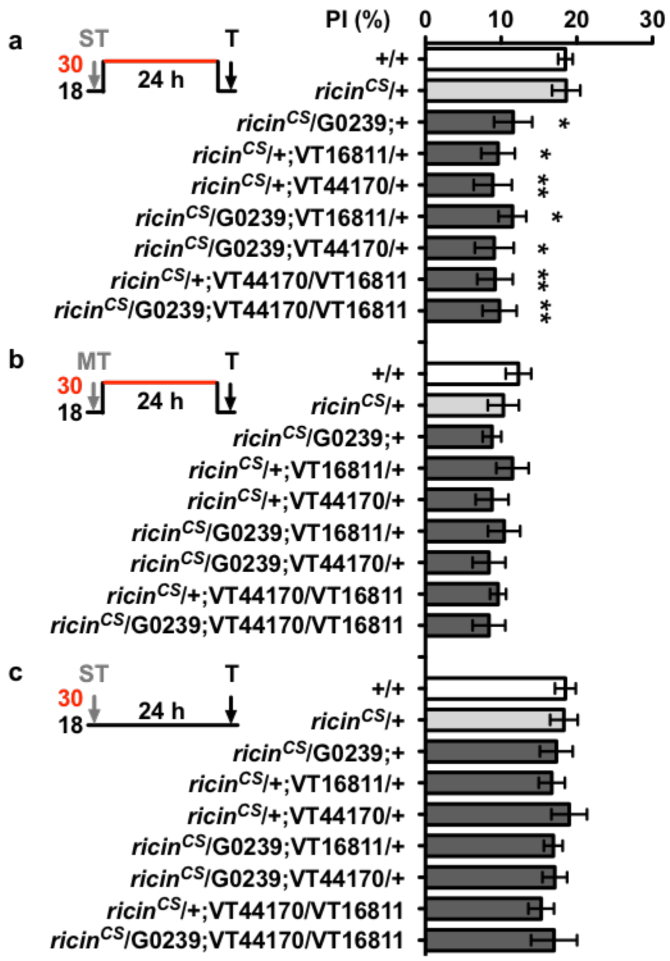

Supplementary Figure 3 | Blocking protein synthesis simultaneously in the three types of MBONs. (**a**) Blocking protein synthesis during consolidation in the combinations of three types of MBON drivers did not cause an additive LTM defect after spaced training (ST), and (**b**) ARMs were all intact after massed training (MT). (**c**) Permissive temperature controls had normal scores of LTM. The drivers used were G0239-*Gal4* for MBON-α3, VT16811-*Gal4* for MBON-γ3,γ3β′1, and VT44170-*Gal4* for MBON-β′2mp. Light gray bar represents effector alone control (*UAS-ricin*^{CS}/+;+) and dark gray bar represents experimental groups. Each value = mean ± SEM ($n \geq 8$). *: $p < 0.05$, **: $p < 0.01$ compared with *UAS-ricin*^{CS}/+;+ effector alone control.

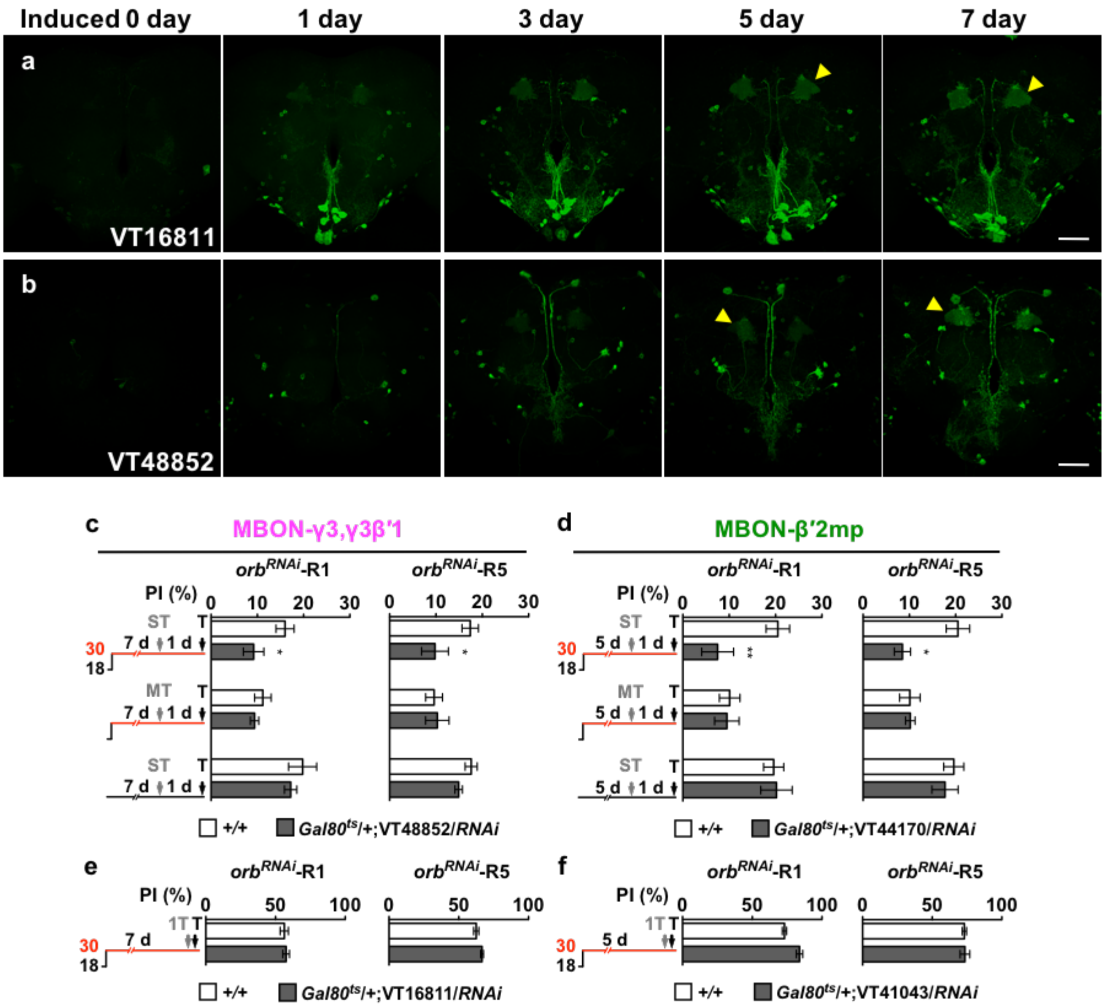

Supplementary Figure 4 | The effects of downregulating ORB for LTM in MBON-γ3,γ3β′1 or MBON-β′2mp independent drivers. (**a–b**) MBON-γ3,γ3β′1 drivers combined with *tub-Gal80^{ts}* in adult flies required a 7-day heat-shock to induce *UAS*-mCD8::GFP expression. The yellow arrowhead indicates the γ3 compartment in the MB. All image data were scanned under the same conditions. Genotypes: *tub-Gal80^{ts}*/*UAS*-mCD8::GFP;VT-*Gal4*/*UAS*-mCD8::GFP. Scale bar: 50 µm. (**c–d**) Flies were trained with spaced training (ST) or massed training (MT) and then tested (T) at 30 °C throughout the experiment. Two *orb* RNAi (R1 or R5) impaired 24-h LTM in VT48852 or VT44170, but not 24-h ARM. Flies of all genotypes under a permissive temperature had normal LTM. Each value = mean ± SEM ($n \geq 8$). *: $p <$

0.05, **: $p < 0.01$. **(e–f)** Downregulating the ORB in MBON-γ3,γ3β′1 or MBON-β′2mp did not affect the learning ability. Learning was performed with one session of training (1T) followed immediately by testing. Each value = mean ± SEM ($n \geq 6$). There were no differences in learning scores among the groups: $p > 0.05$.

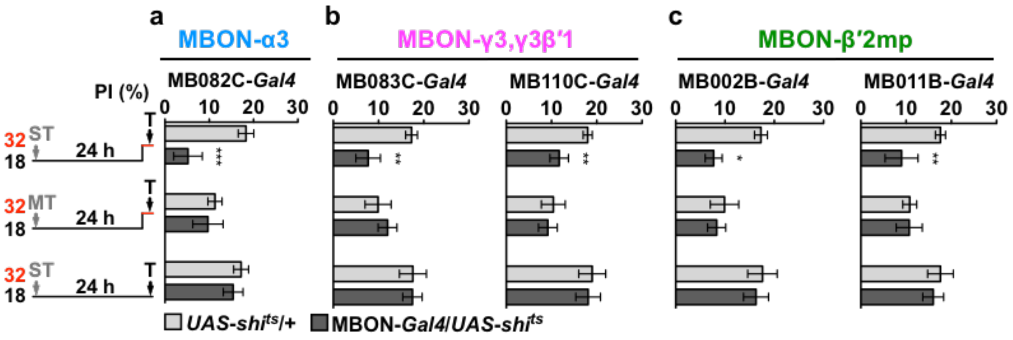

Supplementary Figure 5 | Inactivating neurotransmission output during retrieval in the three types of MBON split-*Gal4* drivers. Flies were trained with spaced training (ST) or massed training (MT) and were then shifted to a restrictive temperature (32 °C) 40 min prior to the test (T). (**a–c**) LTM was impaired by inactivating the neurotransmission output during retrieval in the three types of MBONs, but ARM was intact. Permissive temperature controls showed intact LTM. Each value = mean ± SEM ($n \geq 8$). *: $p < 0.05$, **: $p < 0.01$, ***: $p < 0.001$.

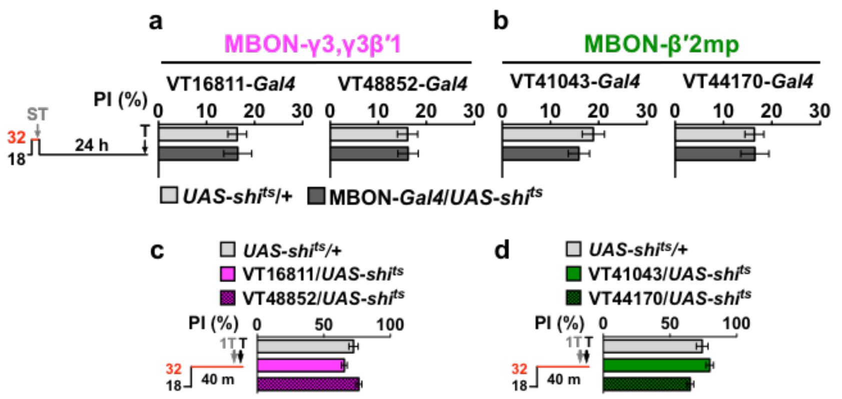

Supplementary Figure 6 | ** Neurotransmission output was not required during training. Inactivating neurotransmission output from MBON-γ3,γ3β′1 (a**) or MBON-β′2mp (**b**) did not affect LTM score during memory acquisition. The flies were acclimated for 40 min and trained with spaced training (ST) at a restrictive temperature (32 °C) to inhibit neurotransmission. After training, the flies were shifted immediately to a permissive temperature (18 °C) and tested (T) 24 h later. Each value = mean ± SEM ($n \geq 8$). There were no differences in LTM scores among the groups: $p > 0.05$. (**c–d**) Inactivating output did not affect learning in MBON-γ3,γ3β′1 or MBON-β′2mp. Learning was performed with one session of training (1T) followed immediately by testing. Each value = mean ± SEM ($n \geq 6$). There were no differences in learning scores among the groups: $p > 0.05$.

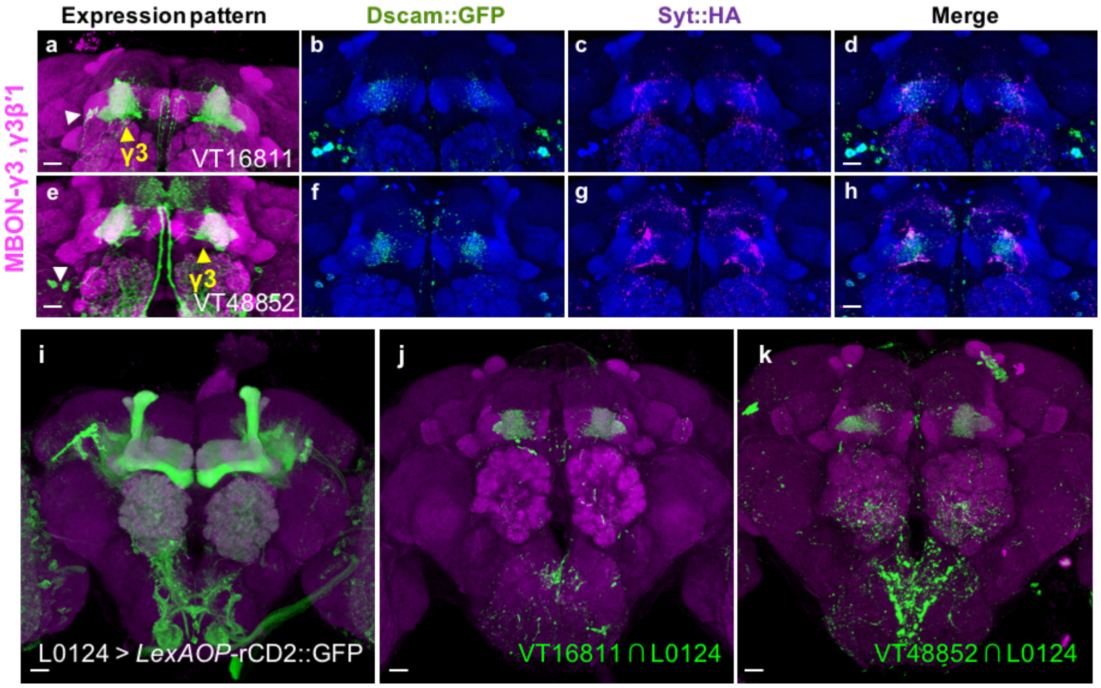

Supplementary Figure 7 | **Survey of the polarity and connectivity of MBON-γ3,γ3β′1. (a,e**) The MB magnified view of MBON-γ3,γ3β′1 drivers. The white arrowhead indicates soma and the yellow arrowhead indicates innervated compartments of MBONs. The whole expression was labeled green, and anti-DLG as a landmark was immunostained magenta. (**b,f**) Dendrites were labeled green by *UAS*-Dscam::GFP, and (**c,g**) axon was labeled magenta by the immunostaining signal of *UAS*-syt::HA. DLG was labeled blue by antibody as a brain landmark. (**i**) Pan-MB driver, L0124-*LexA*, showed whole MB expression in α/β, α′/β′, and γ lobes. (**j–k**) The connectivity of MBON-γ3,γ3β′1 and MB was visualized by GRASP in green. Scale bars for all images: 50 µm.
